# Understanding the complexity of provincial-level elite sport policy change: The case of Shanghai municipality

**DOI:** 10.3389/fpsyg.2022.1044479

**Published:** 2022-10-28

**Authors:** Yang Ma

**Affiliations:** College of Physical Education and Health, Wenzhou University, Wenzhou, China

**Keywords:** elite sport, sport policy, policy change, provincial level, multiple streams framework

## Abstract

The analysis of elite sport policy changes at the provincial level remains relatively uncharted territory despite the substantial contributions of provincial-level elite sport to national elite sport success. Data were gathered from semistructured face-to-face interviews and official and semiofficial documents. The key findings were that (1) Guangdong, as a provincial compatriot of Shanghai, has made tremendous efforts and obtained notable achievements in professional football and thus serves as a powerful stimulant for policy reform regarding elite sports in Shanghai; (2) the policy stream has been strengthened by knowledge-based (epistemic) communities at the Shanghai University of Sports that can examine cause and effect relationships and further propose specific policies; and (3) the general director of the Shanghai Administration of Sports plays a central role in advocating for policy proposals. The current research offers practical insights into strategies for reviewing policy trajectories to enhance policy design and implementation.

## Introduction

Elite sport success has become an ‘*irresistible priority*’ in countries around the world since at least the mid-1960s (Green and Houlihan, 2005). Engulfed by the tide of the ‘*global sporting arms race*’ (Oakley and Green, 2001), elite sport has consistently attracted mainstream policy attention, and policy researchers agree that elite sport success can actually be developed (Grix and Carmichael, 2012; De Bosscher et al., 2015; Zheng and Chen, 2016). Despite the plethora of studies on the static analysis of elite sport policy at the national level (Digel et al., 2003; Green and Houlihan, 2005; Houlihan and Green, 2008) and the growing research attention given to the dynamic analysis of elite sport policy change (Peng et al., 2019; Camargo et al., 2020; Zheng and Liu, 2020), there is a dearth of analysis of elite sport policy and policy change at the provincial level.

This lacuna is surprising considering the substantial support for provincial-level sport organizations and their contributions to elite sport success (Green and Houlihan, 2005; Yamamoto, 2008; Girginov, 2016; Zheng et al., 2018b). Furthermore, the essential role of provincial-level elite sport is described by De Bosscher et al. (2015) as ‘*horizontal coordination at the national level*’ and, equally important, ‘*vertical coordination between the national policy level and provincials*’. In summary, the lack of provincial-level elements in the elite sport policy literature clearly reflects a significant gap in extant elite sport policy research.

China has made great strides in medal tallies at the Summer Olympic Games, with a particularly impressive upswing in gold medals (Brownell, 2008; Tan and Houlihan, 2012; Zheng and Chen, 2016). Sport policy in China examines many themes, including sport for all, professional sport, elite sport, and sport mega events (Zheng et al., 2019). A related recurring question among sport governance and policy researchers is how to analyse and understand Chinese elite sport policy at the national level (Brownell, 2008; Tan and Bairner, 2010; Tan and Houlihan, 2012; Hu and Henry, 2017). Additionally, the first-level administrative divisions (e.g., provinces) have routinely updated their elite sport policy to maintain China’s competitive advantage in these intense medal competitions. It is against this background that the focal policy for this research was planned. On June 6, 2019, the Shanghai Municipal Government (SMG) issued the ‘*Constructing the New Developmental System of Shanghai Elite Sport*’ policy (Shanghai Municipal Government, 2019). This provincial-level elite sport policy is widely considered a general blueprint for the reform of the Shanghai elite sport system.

Hence, this article rests on a case study of Shanghai Municipality, which has not been previously examined by scholars, and fills the abovementioned gap by answering the following research question: How can elite sport policy change in Shanghai Municipality be analysed and understood? Furthermore, in response to Houlihan et al.’s (2009) criticism that meso-level theory is underutilized in studies aiming to advance the understanding of the processes of sport policy making and reform, this research employed the Multiple Streams Framework (MSF) model to fulfil the historical policy narrative of Shanghai elite sport. Subsidiary issues that still need to be clarified to answer the principal research question include the following: What do the problem, policy and political streams contain? Who has played the role of policy entrepreneur?

The remainder of this paper is structured as follows. The next section reviews the literature on Chinese elite sport policy at the national level, the interorganizational relationship literature focusing on provincial-level sport organizations and the MSF put forth by Kingdon (1984). The subsequent methodological section primarily clarifies the research design and the corresponding data collection process. The fourth section presents the main findings. The last section discusses the critical findings, introduces the study’s limitations, and summarizes the research.

## Literature review and theoretical framework

### Chinese elite sport policy at the national level

Research on China has always noted the contrast between the country’s ‘*policy rhetoric*’ and ‘*on-the-ground reality*’ (Yu, 2009). In sport-specific analyses, ‘*sport for all*’ has often been deliberately ignored in favour of elite sport, even though there is a long-standing policy rhetoric aimed at achieving an equilibrium between the two (Zheng et al., 2019). The concept ‘*Juguo Tizhi*’, which can be interpreted as both national and subnational organizations mobilizing their finite resources to bolster elite sport success, emerged following the Sydney 2000 Summer Olympic Games (Brownell, 2008; Hu and Henry, 2017). The enactment of the ‘*Olympic Strategy*’ further legitimized the superior status of elite sport over ‘*sport for all*’ (Zheng et al., 2018a). Furthermore, from the 1990s to 2010s, there were three editions of ‘*The Outline of the Strategic Olympic Glory Plan*’, which steered the progress of elite sport across these three decades (Zheng et al., 2018a; Ma and Kurscheidt, 2019). At a critical juncture (i.e., Beijing’s successful Olympic bid), in addition to a basic policy document (i.e., *The Outline of the Strategic Olympic Glory Plan: 2001–2010*), a specific policy titled ‘*The 2008 Olympic Glory Action Plan*’ was promulgated, highlighting the central government’s pursuit of success on its home soil (Zheng et al., 2018a).

The aforementioned policies to some extent delineate the centralized model of sport governance in China, specifically regarding Olympic elite sport. With the transition from the planned economy era to the reform and opening-up era, Chinese elite sport governance began diverging along two paths of logic: the centralized model of Olympic elite sport and the more liberal and commercialized model of professional football (Zheng et al., 2018a; Xiong and Ma, 2021). This divergence was triggered by an exogenous policy prioritization of ‘*low investment and high return*’ sports or disciplines rather than an endogenous change in market structures (Ma and Kurscheidt, 2020). However, the two logics no longer diverge. Chinese football authorities now seem to be employing a gradualist approach to institutional reform, which greatly facilitates commercialization. To date, only incremental reform at the club governance level has been undertaken (Ma and Zheng, 2022).

### Provincial-level elite sport studies from the interorganizational relationship literature

The existing elite sport policy literature lacks an emphasis on provincial-level elite sport policy (change), even though provincial-level elite sport is regarded as the ‘*backbone and core*’ for delivering elite sport success within the Chinese context (Zheng et al., 2018b). However, this does not mean that there is no concrete research on elite sport development at the provincial level within the Chinese context. Notably, Zheng et al. (2018b) specifically explored interorganizational conflict between national and subnational (provincial) sport organizations in China using three case studies (artistic gymnastics, swimming, and cycling). The study uncovered crucial interorganizational conflicts among national and provincial sport organizations within China’s top-down bureaucratic elite sport logic. Moreover, Ma and Kurscheidt’s (2019) research evaluated the effectiveness of the National Games of China (NGC) in the coordination of public sport governing bodies at the national and provincial levels. The NGC’s character as a ‘governance instrument’ was interpreted in depth through a socioeconomic institutional approach. Despite its relative importance, this line of research provides only limited insights into the policy making of provincial-level elite sport. To date, no in-depth study has examined the policy making process of provincial-level elite sport in the Chinese context. The current research explored the multiple factors influencing the policy making process under the guidance of the MSF.

### Multiple streams framework

De Bosscher et al. (2015) argued that international sporting success is determined by factors at three levels: macro (environment), meso (policy) and micro (talent). A similar point can be made in relation to public policy. It has been suggested that the theoretical realm of public policy can also be categorized into three levels: the macro-, meso-, and micro (Houlihan, 2005). Macrolevel public policy theories refer to the fundamental nature of social and political structures (e.g., pluralism, neopluralism, and elite theory), whereas microlevel policy examines specific decisions or organizations (Houlihan, 2005; Zheng et al., 2018a). Consistent with the central point of this article on elite sport policy changes in Shanghai, it could be argued that the macro- and microlevels are either too broad or too narrow. The mesolevel serves as a heuristic analytical lens that can fulfil the aim of this research, echoing Zheng et al.’s (2018a, 12) assertion that public policy theory at the mesolevel (e.g., the advocacy coalition framework, punctuated equilibrium theory, MSF) is more operational and manageable than that at other levels.

It is worth noting that MSF is one of the most convincing theoretical underpinnings for understanding the crucial timing or juncture of a policy change (Zahariadis, 2007; Weiner, 2011; Baumgartner, 2016). The MSF was developed by Kingdon (1984) to understand the domestic agenda and was derived from the ‘*garbage can*’ model proposed by Cohen et al. (1972), who emphasized ‘*the anarchical nature of organizations and the policy process*’ (Houlihan, 2005). The ‘*garbage can*’ metaphor was further clarified by Cohen et al. (1972) as ‘*various kinds of problems and solutions [being] dumped by participants as they are generated*’. Kingdon (1984, 1995) identified three largely separate streams flowing through the system: the problem stream, the policy stream, and the political stream. These three streams, together with policy windows and policy entrepreneurs, constitute the five structural components of the MSF (Kingdon, 1984, 1995).

The problem stream primarily addresses the specific issues that governments address and attempt to resolve. These problems are not always identifiable (Peng et al., 2019), but government policymakers generally identify problems *via* three approaches: indicators, focusing events, and feedback on the effectiveness of existing policies (Houlihan, 2005). First, indicators refer to the assessment of the severity of a problem. In the elite sport policy area, the decline in the number of gold medals at the Summer Olympic Games rendered the problem visible. Second, focusing events, notably crises or disasters, draw governments’ attention to detailed issues. Finally, problems can also be identified through feedback on the performance of current policies (Houlihan and Lindsay, 2013; Salisbury, 2016).

Analogous to ‘*primeval soup*’ (Parsons, 1995), the term policy stream refers to ‘*ideas, backed by particular policy communities, float around and occasionally combine and rise to the top of the agenda*’ (Houlihan, 2005, 171). The process by which ideas compete for acceptance and legitimacy is akin to that of natural selection. Selected and accepted policy ideas may rise to the top of the agenda, while the rest remain at the bottom (Wu, 2021). In addition, two criteria influence why some ideas gain prominence and others are neglected: technical feasibility and value acceptability (Zheng et al., 2018a, 27). The latter refers to consistency with the community’s dominant values (Houlihan, 2005).

The political stream basically includes three components: the national mood, organized political forces, and the government. In line with Zahariadis (2007, 73), the national mood can be conceptualized as ‘*a fairly large number of individuals in a given country tend[ing] to think along common lines and… the mood swing[ing] from time to time*’. The second influencer proposed by Kingdon (1984, 1995) is organized political forces, such as parties and interest and pressure groups. It is worth noting that the second influencer is less likely to actualize in the Chinese context, which is characterized by a one-party political system (Peng et al., 2019, 5). Furthermore, echoing Zahariadis’s (2007) argument, the national mood and the third influencer (i.e., government) are highly likely to influence government agendas. The last influencer is defined as the key personnel and structural changes within the government (Zahariadis, 2007; Zheng and Liu, 2020).

Policy windows provide chances for proponents to initiate action to advocate for their selected ideas or draw attention to specific issues (Wu, 2021). Kingdon (1984, 1995) identified two types of policy windows, namely, problem windows and political windows. This classification indicates the unfolding mechanisms of a policy window that can be facilitated by a problem stream or a political stream. Moreover, although the three streams are largely separate, they can merge at critical junctures. In line with Parsons’s (1995, 194) conclusion, ‘*if all three streams are joined then the item has a high probability of reaching the top of the decision agenda*’.

The abovementioned coincidence of the three streams requires a facilitator (Zheng et al., 2019) or a broker (Ackrill and Kay, 2011) to sell the ideas or proposals to policy makers. Policy entrepreneurs are ‘*advocates who are willing to invest their resources to promote a position in return for anticipated future gain in the form of material, purposive, or solidary benefits*’ (Kingdon, 1984, 1995). Policy windows are of short duration, and policy entrepreneurs feel obliged to make the most of the hard-earned opportunity to advocate for their agenda. The indefatigability of policy entrepreneurs as advocates is underlined. The success of policy entrepreneurs relies significantly on their capacity to look for relevant policy makers who are receptive to their intentionally selected proposals (Salisbury, 2016).

As a major theoretical breakthrough in the study of public policy (Sabatier, 1999), the application of the MSF has grown considerably in recent years in terms of geographical and cultural spread (e.g., Chalip, 1996; Richardson, 2001; Travis and Zahariadis, 2002; Teodorovic, 2008; Lancaster et al., 2012). Its effectiveness in the Chinese context has been fully proven by, for instance, policy-making studies on the Chinese detention and repatriation system (Zhu, 2008) and on Chinese college matriculation (Zhou and Feng, 2014). More specifically, in relation to sport policy analysis in the Chinese context, MSF’s applicability has also been demonstrated, as exemplified by studies focused on Chinese elite swimming policy changes between 2000 and 2012 (Zheng, 2017), the analysis of the Chinese football reform of 2015 (Peng et al., 2019), and policy analysis of elite sport development in Hong Kong (Wu, 2021). In summary, the MSF can be perceived as a useful theoretical perspective for analysing Shanghai’s elite sport policy.

## Research methods

### Research paradigm and research design

The research paradigm was conceptualized by Bailey (1994, 26) as ‘*the metal window through which the researcher views the world*’. It exerts considerable influence on the researcher’s thinking regarding the world and his or her interpretation of social phenomena (Grix, 2010; Wu, 2021). This research followed the ‘critical realist’ approach put forth by Bhaskar (1989). Different from realists’ assertion that social phenomena have objective attributes and exist outside of human influence (Bryman, 2016) and from constructivists’ argument that social phenomena and their corresponding meanings are constantly being performed by social actors (Bryman, 2016), critical realists’ position occupies a middle ground on this spectrum, with realists at one end and constructivists at the other, advocating that the social world can be understood only by recognizing the structures that call into being events and discourses, and the identification of the structures can be fulfilled only by means of the practical and theoretical work of the social sciences (Bhaskar, 1989).

Hence, critical realism argues that social facts need to be objectively reflected. Nevertheless, since not all social facts can be observed and quantified, it is critical to embrace the subjective element. According to Guba and Lincoln (1994), knowledge of the subject under investigation (i.e., elite sport policy change at the provincial level) is believed to exist but is only ‘*imperfectly apprehendable because of basically flawed human intellectual mechanisms and the fundamentally intractable nature of phenomena*’. Therefore, this study employed a qualitative strategy, counting on *‘nonnumerical analysis to provide understanding*’ (Gratton and Jones, 2010).

This research primarily used a single-case study design (Yin, 2009). The MSF is used to structure a case study of the 2019 Shanghai elite sport policy reform. In line with the argument of Travis and Zahariadis (2002, 495), MSF researchers adopt qualitative case study approaches in a typical manner, and Kingdon’s work has rarely been used in quantitative studies. Three criteria for case selection reveal why the research focuses on Shanghai Municipality: This case is significant (Denscombe, 2007) and representative (Stake, 1995) or exemplifying (Bryman, 2016) and presents a convenient and feasible study object (Denscombe, 2007). Shanghai, as the economic centre of the People’s Republic of China (PRC), occupies an indispensable position within the Chinese elite sport landscape, as strongly evidenced by the fact that Yao Ming, who has been hailed as ‘*an icon of confidence for China*’ (Xu, 2008, 210), and Liu Xiang, who brought the PRC its first Olympic gold medal in men’s track and field, both came from Shanghai. In addition, the case selection was facilitated by the researchers’ connections within the Shanghai elite sport system, ability to speak both Chinese and English, and strong knowledge of official and semiofficial document sources.

Notably, this research does not endeavour to offer an ‘*all-powerful formula*’ (Zheng et al., 2018b), ‘*one-size-fits-all*’ solution (Cloete and De Coning, 2016) or ‘*silver bullet*’ (Wu, 2021) that is useful to all nations or regions. Instead, it focuses on ‘thick description’ (Tracy, 2010) in the context of Shanghai Municipality, which is in line with the argument that contextual uniqueness is an inherent feature of social phenomena and accompanying qualitative research (Shenton, 2004; Tan et al., 2019). Therefore, audiences both within and outside academia are encouraged to evaluate the transferability of the results based on their specific situations in a critical manner.

### Data collection

This study adopted a qualitative method. Considering the value of primary interview data (Miller and Sinanan, 2014), semistructured face-to-face interviews were conducted even though the COVID-19 outbreak was spreading. This is because in mainstream Chinese culture, in the minds of elderly and middle-aged persons, non-face-to-face interviews are impolite and make them reluctant to share their thoughts. Using *a ‘judgemental sampling strategy*’ (Blaikie, 2010) or *‘purposive sampling strategy*’ (Abrams, 2010), first-hand materials from related stakeholders were collected. Table 1 presents details (alias, organization and position) on the four interviewees.

**Table 1:** Profiles of the interviewees.

Interviewee ID	Age	Organization	Position
A	38	Shanghai Administration of Sports	Deputy director responsible for elite sport
B	35	Sports Bureau of Yangpu District	Director responsible for elite sport training
C	59	Shanghai University of Sport	Professor specializing in elite sport
D	43	Shanghai University of Sport	Professor specializing in sport policy

All interviewees were informed about the essence of the research, a process that drew upon a translated Chinese version of an ethics checklist and included soliciting the participants’ signed informed consent. Generally, the interview protocol employed in this study was tightly focused on the research question mentioned in the introduction section. The semistructured face-to-face interviews lasted between 30 min and 1 h each.

Perceived as ‘*windows onto social and organizational realities*’ (Bryman, 2016), relevant official and semiofficial documents supplement the semistructured interviews. Official (e.g., from the Shanghai Administration of Sports and Shanghai University of Sport) and semiofficial (e.g., from the websites of Oriental Sports Daily and Great Sports) documents were collected for secondhand data. Additionally, peer-reviewed academic articles in Mandarin from the Chinese National Knowledge Infrastructure, i.e., the Chinese version of the Web of Science, were examined.

### Data analysis and trustworthiness

The discrepancies between official and semiofficial documents and the collected interview transcripts were scrupulously cross-checked. The interview transcripts were carefully examined to resolve any inconsistencies with audio recordings. Then, the interview transcripts were shared with the corresponding interviewees for further validation (Braun and Clarke, 2006; Burke, 2016). With regard to the non-English data, back translation was used to guarantee the data quality. The thematic analysis approach was used to fix the qualitative data (Boyatzis, 1998; Guest et al., 2011). More specifically, on the one hand, deductive coding processes and theme identification were achieved in accordance with the well-defined fundamental elements of the MSF (Ryan and Bernard, 2003); on the other hand, inductive coding approaches focused on the codes derived directly from the data, enabling me to interpret phenomena that could not be explained by existing theories or concepts (Miles and Huberman, 1994; Fereday and Muir-Cochrane, 2006).

## Findings

### Problem stream

As the first structural element of the MSF, the problem stream was conceptualized as the underlying rationale that the government followed to launch the policy change. In line with the MSF put forth by Kingdon (1984, 1995), three factors were considered to identify problems: focusing events and crises (e.g., poor performance), indicators, and feedback on the effectiveness of existing policies. Regarding the ‘*focusing events and crises*’, a director inside the Sports Bureau of Yangpu District made the following comment:

Everyone within the elite sport system knows that the NGC is the most important sport event in domestic China. Actually, it is a platform in which every province ‘fights’ or competes with each other, signalling its economic and cultural power. Unfortunately, the sporting performance of Shanghai at the NGC cannot match its role as an economic pioneer in domestic China (Interviewee B).

As presented in the literature review section, the NGC is widely considered the ‘*Olympic Games of China*’, mainly due to its identical format to the Olympic Games and its prominent national status (Ma and Kurscheidt, 2019). In essence, the NGC can be regarded as a concrete platform for first-level administrative divisions to compete with each other (Zheng et al., 2018b). The Shanghai delegation has not ranked in the top two in the medal tally since the 9th NGC. The sporting performance of the Shanghai delegation within the NGC platform has been continually criticized for its mismatch with its role as an economic pioneer (Miao, 2012, 2013). Furthermore, the acquisition of medals heavily relies on the introduction of elite athletes from other provinces. A market mechanism, i.e., a pricing of talent, was introduced into the elite sport system following a top-down logic (Ma and Kurscheidt, 2019). In terms of ‘*indicators*’, this mechanism can be applied to assess the magnitude of change in the hope of catching official attention (Travis and Zahariadis, 2002). At the 9th NGC, 14% of all gold medals obtained by the Shanghai delegation were won by elite athletes from other countries (Miao, 2012, 2). This figure increased to 38 and 36% at the 10th and 11th NGCs, respectively (Miao, 2012, 2), and this phenomenon attracted close attention from the SMG. Interviewee A asserted the following:

SMG, to some extent, was not satisfied with our sporting performance at the NGC platform recently. It seems that we poured a large sum of money into the introduction of elite athletes from other provinces. To be honest, this is a shorter route than the usual one. This kind of shortcut will definitely exert a negative influence on the sustainability of elite sports in Shanghai (Interviewee A).

Shanghai’s constantly poor performance, the mismatch between its sporting performance and its role as an economic pioneer, and the municipality’s heavy reliance on the introduction of elite athletes from other provinces are identified as a source of problems. In contrast to the common belief that negative focusing events such as poor performance are more likely to facilitate policy change (Jones, 1994), this research revealed that the stunning performance of provincial compatriots was more likely to reveal the policy window, as was confirmed by Interviewee B:

Football was the first sport to be professionalized in China. The development of professional football has attracted increased attention in wider society. Guangzhou Evergrande brings great honour to Guangdong Province. The club wins the toptiered title each year from 2011 to 2017. This club was reputed as the only one that has ever won the Asian Champions League. By stark contrast, Shanghai professional football is losing its lustre. From the standpoint of SMG, it is a big problem (Interviewee B).

The tremendous pressure exerted by provincial compatriots, i.e., Guangdong Province, was reconfirmed by document sources. Miao (2012, 2) asserted that Shanghai experiences is pained byGuangdong Province’s excellent performance in professional football because the latter’s impressive advancement in professional football causes Shanghai to be overshadowed. In particular, in the Asian Football Confederation (AFC) Champions League, clubs from Guangzhou managed to defeat Korean and Japanese clubs on behalf of the PRC after President Xi Jin-Ping highlighted his dreams for the country in regard to football.

With regard to the last factor, ‘*feedback on the effectiveness of existing policies*’, Interviewee D clarified that the policy was improper for the occasion, as was also confirmed by Interviewee C.

The old elite sport policies have always stressed the crucial and indispensable role that SMG plays. It is common sense that SMG shoulders the responsibility to distribute financial and human resources. To some extent, the old elite sport policy has doubts about the involvement of social forces. However, it is absolutely crazy for the time being that we exclude social forces from the Shanghai elite sport landscape (Interviewee D).

Social progress brings a considerable increase in training expenditures and travelling costs. Currently, the training expenditures are 10 times higher than in the old days. The travelling cost is even 50 times [higher]. Elite sport has evolved into a huge burden for our government’s overhead. We need to reform elite sport policy, inviting the deep involvement of social forces (Interviewee C).

In line with Zheng et al. (2019), Chinese sport policy embraces the contrast between a politically led approach as a relic of the planned economy and a market-led approach. Notably, the shortage of financial resources is more obvious in money-consuming disciplines (e.g., equestrianism and golf) and newly added Olympic disciplines (e.g., rock climbing and breakdancing; Ma and Kurscheidt, 2021) than in other disciplines. In addition, in relation to the effectiveness of existing policies, the lack of emphasis on nurturing world-famous sport stars has been noted and firmly criticized.

Oriented as an international well-known mega-city, Shanghai is obliged to nurture internationally renowned sports stars. We should not immerse ourselves in the old times when we have Yao Ming and Liu Xiang. For the time being, we have nothing. Fortunately, the SMG realized that imperative policy inference is of great importance for nurturing sports stars (Interviewee D).

**Figure 1:**
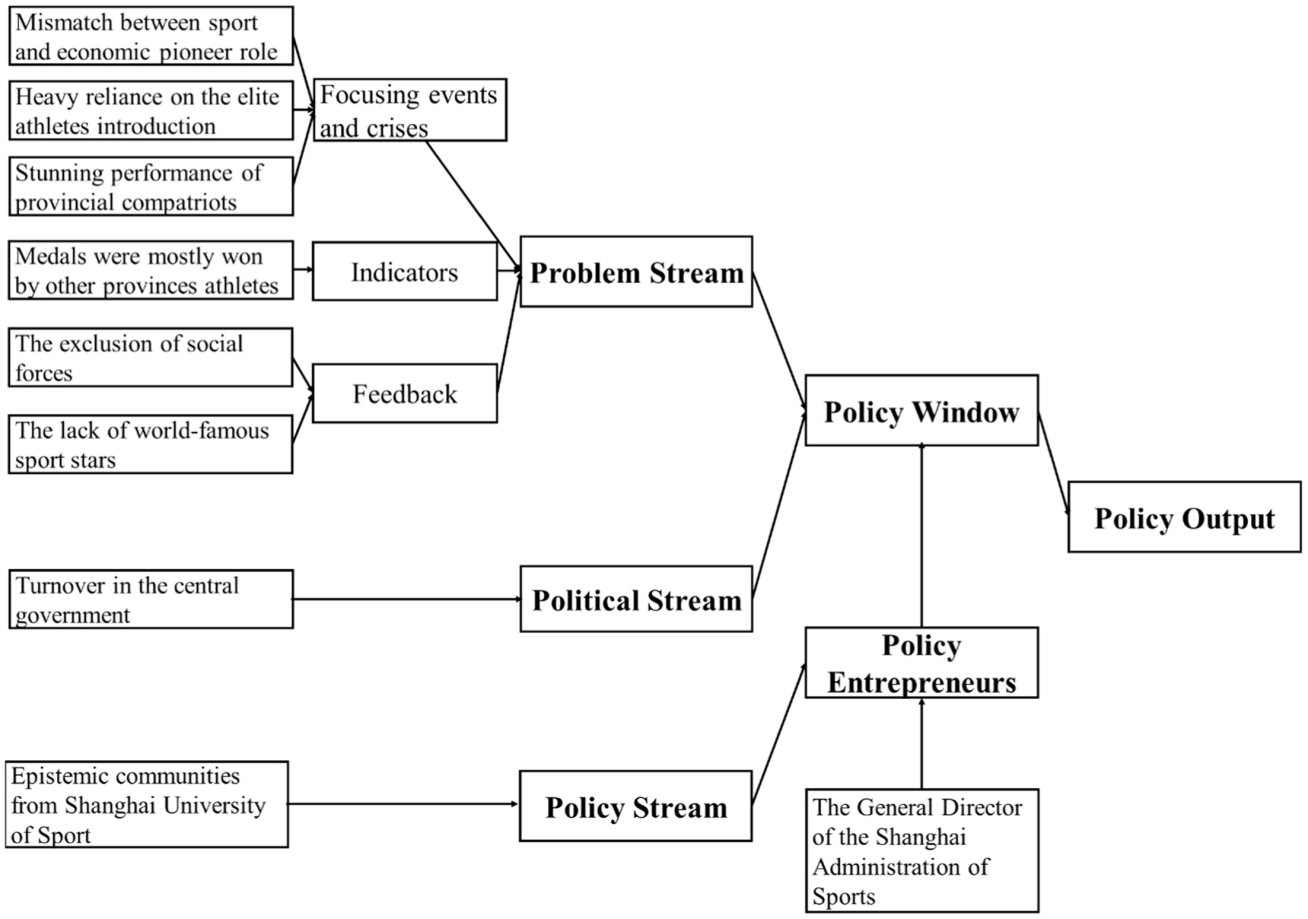
Schematic diagram of the theoretical framework.

To summarize, as shown in Figure 1, the problem stream comprises the following factors: focusing events and crises (the mismatch between Shanghai’s sport performance and its role as an economic pioneer, the heavy reliance on the introduction of elite athletes from other provinces, and the stunning performance of provincial compatriots), indicators (a large percentage of the total gold medals obtained by the Shanghai delegation were won by elite athletes from other countries), and feedback on the effectiveness of existing policies (the exclusion of social forces from the Shanghai elite sport landscape and the lack of emphasis on nurturing world-famous sport stars).

### Policy stream and policy entrepreneurs

The policy stream can be interpreted as a simplified ‘*primeval soup*’ of ideas (Parsons, 1995). Ideas are advocated by policy communities that are composed of specialists. Policy communities were described by Kingdon (1984, 1995) as performing either individually or together to fulfil shared goals. However, for this research, the notion of an ‘*epistemic community*’ developed by Haas (2009) is more suitable because the set of alternatives to the governmental agenda was initiated by scholars at Shanghai University of Sport, as was confirmed by Interviewee D:

As the only sport-specific university in Shanghai Municipality, Shanghai University of Sport shouldered the responsibility to draft a set of alternatives to the governmental agenda. Several professors specializing in elite sport and elite sport policy were selected for intellectual thinktanks (Interviewee D).

The term ‘*epistemic community*’, or ‘*intellectual thinktanks*’, which refers to a network of professionals with renowned expertise and competence in a specific area (Haas, 2009, 3), is beneficial for understanding the policy stream considered in this research. Of particular importance is that the knowledge-based (epistemic) communities from Shanghai University of Sports can examine cause-and-effect relationships and further propose specific policies.

Policy entrepreneurs are eager to advocate their favoured ideas among policy communities and even wider audiences with the aim of increasing the acceptance of their ideas. Echoing Peng et al.’s (2019) and Wu’s (2021) assertions, I noted that policy entrepreneurs from mainland China are always characterized by bureaucratic attributes. In this research, the general director of the Shanghai Administration of Sports is recognized as playing a central role in policy making. Elite sport policy reform is a highly sought-after outcome for the general director of the Shanghai Administration of Sports. Interviewee C clarified the underlying rationale for this role:

Our general director has the greatest personal motivation to play a central role and facilitate policy change in Shanghai elite sport, pushing the sporting performance of the Shanghai delegation at the NGC platform to a higher level. The underlying rationale is that the medals obtained from the NGC, particularly, the gold, have a direct relationship with job promotion. Fully immersing in the policy reform of elite sport is his normal routine (Interviewee D).

### Political stream

The political stream involves factors creating an environment beneficial to agenda change (Ackrill and Kay, 2011). Kingdon (1984, 1995) illustrated the political stream as having the following three influencers: the national mood, organized political forces, and the government (legislative and administrative turnover). Within the Chinese context, there is no democratic tool equivalent to the public referendums seen in Western societies to determine whether the national mood towards elite sport development is positive or negative. In a similar vein, the CPC will never permit organized political forces to exert any impact on the governmental agenda (Zheng, 2017; Peng et al., 2019). With regard to the third element of the political stream, the ascension of a new president or new secretary of a state implies potential changes (Travis and Zahariadis, 2002). Turnover in the central government strongly influences the SMG’s willingness to initiate policy changes in elite sport, *inter alia*, in the area of professional football (Interviewee C).

The SMG is obliged to make prompt, positive and concrete responses to President Xi’s three wishes for Chinese football: participating in the World Cup, hosting the World Cup, and being World Cup champions. Professional football is regarded as an effective tool for facilitating the formation of the talent pool. The SMG is eager to reform the old elite sport system, especially in the area of professional football (Interviewee C).

## Discussion

Much of the extant research in the field of sport management and sport policy has focused on elite sport policy at the national level (Piepiora, 2019), which has created a gap in the understanding of the reform process of elite sport policies at the provincial level. In addition, most discussions of the policy reform of Shanghai elite sport are predicated on a single, salient casual event, the persistent underachievement of the Shanghai delegation in the NGC platform or the pursuit of rapid success, which could short-circuit the development of Shanghai elite sport. Clearly, no individual component is sufficient for fully interpreting the policy reform. The contribution of the MSF to this case study was to draw together these elements in the policy window. The MSF is well-suited for studying Shanghai’s elite sport policy, which further supports the decontextualization process of the model. First, the problem stream includes concerns that draw the attention of policy-makers (Ackrill and Kay, 2011). For example, the problem stream comprises the following factors: focusing events and crises (the mismatch between Shanghai’s sport performance and its role as an economic pioneer, the heavy reliance on the introduction of elite athletes from other provinces, and the stunning performance of provincial compatriots), indicators (a large percentage of the total gold medals obtained by the Shanghai delegation were won by elite athletes from other provinces), and feedback on the effectiveness of existing policies (the exclusion of social forces from the Shanghai elite sport landscape and the lack of emphasis on nurturing world-famous sport stars).

The policy stream is where ideas and proposals are formulated and revised (Ackrill and Kay, 2011). In the present case, this stream is strengthened by knowledge-based (epistemic) communities from the Shanghai University of Sports, which share a common concern in a single policy area (elite sport) and can examine cause-and-effect relationships and further propose specific policies. The general director of the Shanghai Administration of Sports is recognized as playing a central role in advocating for policy proposals. This finding echoes Ackrill and Kay’s (2011) and Bakir and Jarvis’s (2017) argument that it is unlikely that a single person can be solely responsible for such a change. However, it is arguable that individuals can play a central role in the policy change process.

The political stream is mainly manifested as governmental influence (legislative and administrative turnover). Turnover in the central government has strongly influenced the SMG’s willingness to initiate policy change in elite sport, particularly in the area of professional football. Considered collectively, the problem, policy and political streams have emerged and combined into a single unit with the support of policy entrepreneurs, which significantly increases the possibility of a consensus regarding policy change (reform). With the opening of the policy window, rather than simply being pushed into obscurity, the issue of elite sport policy change has reached the top of the decision agenda in Shanghai and received serious attention from policymakers. Kingdon (1984, 1995) clarified the difference between decision agendas and governmental agendas. When a policy is on the decision agenda, it has a more active status than when on the governmental agenda.

Ultimately, this research both makes academic contributions and has practical utility. In relation to academic contributions, although the MSF, which was introduced by Kingdon in the United States, is being increasingly adapted to the study of Chinese policy-making, this research has revealed a theoretical underdevelopment in some of its central components. Theoretical development was first achieved by enriching the content of the ‘*focusing events and crises*’ factor by adding the overshadowing performance of provincial compatriots. Guangdong, as a provincial compatriot of Shanghai, has exerted tremendous effort and made notable achievements in professional football, which acts as a powerful stimulant for and has facilitated the policy change process for elite sport in Shanghai. Second, in the application of the MSF to Shanghai studies, it is argued that compared with the broader concept of ‘*policy community*’, the term ‘*epistemic community*’ is much more appropriate in this study. This adaptation is relevant to the present case study. However, this research’s support for the ‘*epistemic community*’ comes with the caveat that our case centres on knowledge-based communities from the Shanghai University of Sports. The final theoretical contribution offered refers to the term ‘policy entrepreneur’; Ackrill and Kay’s (2011) unambiguous distinction between individuals who are policy entrepreneurs and the process of policy entrepreneurship may have some implications for this research. The general director of the Shanghai Administration of Sports can act as a policy entrepreneur in selling a policy proposal, but he or she may not be aligned with Kingdon’s (1995) construct of a policy entrepreneur due to his or her institutional role. In the Chinese context, participants who hold formal positions always play a crucial role in the decision-making process (Peng et al., 2019, 6). Hence, in our case, policy entrepreneurship is understood as a temporary characteristic that is context- and situation-specific and is considered a dynamic entity with the potential for continual changes over time. In practical terms, from the standpoint of policy makers in Shanghai, this research provides a valuable opportunity to review the corresponding policy trajectory to improve the effectiveness and efficacy of existing policies.

It is worth noting that this research has several limitations, but it also offers directions for future research. First, this research employed qualitative techniques and focused on ‘thick description’ (Tracy, 2010) and the uniqueness of the *broader distal environment* (Chelladurai, 2014). I recognize that this method is potentially limited in its ability to generalize our findings (Gratton and Jones, 2010) and offer an ‘*all-powerful formula*’ (Zheng et al., 2018b), ‘*one-size-fits-all*’ solution (Cloete and De Coning, 2016) or ‘*silver bullet*’ (Wu, 2021) that is useful to all other nations or regions. Nevertheless, audiences both within and outside academia are encouraged to evaluate the transferability of the results by further analysing the intricacies of each context.

Second, in line with the argument made by Barzelay and Gallego (2006), this research uses problem, policy and political streams as descriptive heuristics to aid the organization of a historical policy narrative. Further studies are encouraged to probe into the nature and duration of policy spillover within multiple institutionally connected policy fields. Unfortunately, Kingdon (2003, 190) mentioned only the exogenous spillover between institutionally unrelated policy areas and thus seriously neglected the endogenous spillover supplemented and elaborated by Ackrill and Kay (2011) in their analysis of sugar reform in EU policy-making. Interestingly, the endogenous spillover between multiple institutionally connected policy areas can manifest in policy windows that are open for longer periods of time. This argument, to some extent, explains the relatively delayed sport policy reform in contrast to the prompt action in economic areas within the Chinese context.

Third, the PRC, which is the world’s second-largest country by land area, covers approximately 9.6 million square kilometres with a population of over 1.35 billion people and is structured into 22 provinces, 5 autonomous regions (Guangxi Zhuang, Inner Mongolia, Ningxia Hui, Tibet and Xinjiang Uygur), 4 directly controlled municipalities (Beijing, Chongqing, Shanghai and Tianjin) and 2 special administrative regions (Hong Kong and Macau). The majority of these jurisdictions each have populations of approximately 10 million people. Some of these jurisdictions have fewer than 9 million inhabitants (Hainan, Ningxia, Qinghai and Tibet), whereas other provinces (Anhui, Guangxi, Shandong, Yunnan and Zhejiang) have over 45 million citizens (National Bureau of Statistics, 2010). Provincial-level sport research holds intriguing academic potential in examining regional diversity, administrative complexity and the significant gaps in the sport policy and management literature. This finding highlights the need for more in-depth research on provincial-level sport development.

## Data availability statement

The original contributions presented in the study are included in the article/Supplementary material, further inquiries can be directed to the corresponding author/s.

## Ethics statement

Ethical review and approval was not required for the study on human participants in accordance with the local legislation and institutional requirements. Written informed consent for participation was not required for this study in accordance with the national legislation and the institutional requirements.

## Author contributions

The author confirms being the sole contributor of this work and has approved it for publication.

## Funding

This work was supported by the China Postdoctoral Science Foundation (No. 2022M711426).

## Conflict of interest

The author declares that the research was conducted in the absence of any commercial or financial relationships that could be construed as a potential conflict of interest.

## Publisher’s note

All claims expressed in this article are solely those of the authors and do not necessarily represent those of their affiliated organizations, or those of the publisher, the editors and the reviewers. Any product that may be evaluated in this article, or claim that may be made by its manufacturer, is not guaranteed or endorsed by the publisher.
